# Nonsense-mediated decay as a terminating mechanism for antisense oligonucleotides

**DOI:** 10.1093/nar/gku184

**Published:** 2014-04-03

**Authors:** Amanda J. Ward, Michaela Norrbom, Seung Chun, C. Frank Bennett, Frank Rigo

**Affiliations:** Department of Core Antisense Research, ISIS Pharmaceuticals, Inc., Carlsbad, CA 92010, USA

## Abstract

Antisense oligonucleotides (ASOs) are synthetic oligonucleotides that alter expression of disease-associated transcripts via Watson–Crick hybridization. ASOs that function through RNase H or the RNA-induced silencing complex (RISC) result in enzymatic degradation of target RNA. ASOs designed to sterically block access of proteins to the RNA modulate mRNA metabolism but do not typically cause degradation. Here, we rationally design steric blocking ASOs to promote mRNA reduction and characterize the terminating mechanism. Transfection of ASOs complementary to constitutive exons in *STAT3* and *Sod1* results in greater than 70% reduction of mRNA and protein. The ASOs promote aberrant exon skipping and generation of premature termination codon (PTC)-containing mRNAs. We inhibit the nonsense-mediated mRNA decay (NMD) pathway and show that the PTC-containing mRNAs are recognized by the UPF1 ATPase, cleaved by the SMG6 endonuclease and degraded by the XRN1 cytoplasmic exonuclease. NMD surveillance, however, does not entirely explain the mechanism of decreased *STAT3* expression. In addition to exon skipping, ASO treatment causes intron retention and reduction of chromatin-associated *STAT3* mRNA. The application of steric blocking ASOs to promote RNA degradation allows one to explore more nucleotide modifications than tolerated by RNase H or RISC-dependent ASOs, with the goal of improving ASO drug properties.

## INTRODUCTION

Antisense oligonucleotides (ASOs) are powerful and extremely versatile therapeutic agents utilized in a growing number of applications including RNA reduction, translation arrest, miRNA inhibition, splicing modulation and polyadenylation site selection (1,2). The most widespread use of ASOs is for decreasing the expression of protein coding RNAs through an RNase H or RNA-induced silencing complex (RISC) mechanism. ASOs containing DNA recruit RNase H to the DNA–RNA heteroduplex, where it cleaves the RNA and promotes subsequent degradation by cellular nucleases (3). In contrast, small interfering RNAs (siRNAs) reduce mRNA expression after assembly of RISC and Argonaute 2 (AGO2) cleavage of the target mRNA (4).

A chemical modification strategy is crucial for improving the stability, tissue distribution and hybridization affinity of unmodified nucleic acids. Adequate pharmacokinetic and pharmacodynamic drug properties can be attained by the incorporation of backbone modifications (such as phosphorothioate) and 2′-sugar modifications (such as 2′-*O*-methoxyethyl, MOE) to decrease nucleolytic degradation and increase binding affinity (5). Unfortunately, 2′-modifications in the central DNA ‘gap’ of an ASO impair RNase H activity and are therefore limited to the 5′ and 3′ wings of the ASO (6). Similarly, single-stranded and double-stranded siRNAs have unique structural and chemical requirements that limit the repertoire of modifications supportive of AGO2 activity (7,8).

In contrast to ASOs that function through an enzymatic mechanism, single-stranded ASOs that sterically block access of RNA binding proteins to the RNA are amenable to chemical modifications at every nucleotide position. Steric blocking ASOs have frequently been used to modulate mRNA metabolism, including splicing correction applications (2,9). Here, we ask if steric blocking ASOs can achieve mRNA reduction by exploiting endogenous cellular surveillance pathways that recognize and degrade aberrant mRNAs. One such pathway is nonsense-mediated mRNA decay (NMD), which modulates gene expression and prevents mRNAs from producing potentially toxic proteins.

Defects in pre-mRNA processing can result in protein loss-of-function when a premature termination codon (PTC) disrupts the open reading frame. As a general rule, when a PTC is located greater than 55 nucleotides upstream of the last exon–exon junction, the spliced PTC-containing mRNA is a likely substrate for NMD (10). In brief, this process involves communication between the translating ribosome and components of the exon junction complex, including the essential NMD factor UPF1, to degrade the RNA by endonuclease and exonuclease activity (11,12).

Fully modified morpholino ASOs (13,14) and 2′-*O*-methyl ASOs (15), not supportive of RNase H or RISC activity, targeted to splicing regulatory elements achieved mRNA reduction by disrupting the transcript's open reading frame. While it is hypothesized that these ASOs utilized the NMD pathway, this has not been explicitly examined. In addition, no studies have directly compared ASO-mediated target reduction through NMD-dependent and RNase H-dependent mechanisms.

As proof of concept that ASOs can rationally be designed to cause mRNA reduction by directing transcripts to the NMD pathway, we synthesized steric blocking ASOs complementary to specific constitutive exons in human *STAT3* and mouse *Sod1*. In theory, skipping of *STAT3* exon 6 and mouse *Sod1* exon 2 or exon 3 would result in a frameshift and generation of PTC-containing mRNAs. After identifying ASOs that disrupted proper pre-mRNA processing, we characterize for the first time the molecular pathway leading to target reduction. Moreover, we compare the potency of ASOs that act through NMD-dependent and RNase H-dependent mechanisms *in vitro* and *in vivo*.

## MATERIALS AND METHODS

### Oligonucleotide synthesis

Synthesis and purification of all chemically modified oligonucleotides was performed as previously described (16).

### Cell culture, siRNA and ASO transfections, and emetine treatment

HeLa (CCL-2) and bEnd.3 (CRL-2299) cells were maintained according to American Type Culture Collection (ATCC) standards. For siRNA transfection, cells were plated and 24 h later transfected with 10 nM individual siRNA (Thermo Scientific) using Lipofectamine RNAiMAX (Life Technologies) for 48 h according to the manufacturer's instructions. ASO transfections were performed 24 h after cell plating or 48 h after siRNA transfection. Fifty nanomolars ASO was transfected using 3 μg/ml Cytofectin transfection reagent for 24 h. For translation inhibition experiments, emetine (10 μg/ml prepared in H_2_O) or H_2_O was added to the culture medium 20 h after ASO transfection and remained on the cells for 4 h. Cells were then washed and lysates prepared for analysis. See Supplementary Tables S1 and S2 for ASO and siRNA sequences, respectively. Silencer Select Negative Control No.1 siRNA (Life Technologies) was used as the negative control siRNA in all experiments.

### Standard RT-PCR and quantitative RT-PCR

Total cellular RNA was isolated using the RNeasy kit (Qiagen). For standard reverse transcriptase-polymerase chain reaction (RT-PCR), 400 ng RNA was reverse transcribed using random hexamers and SuperScript II Reverse Transcriptase (Life Technologies) followed by PCR with Platinum Taq PCR Polymerase (Life Technologies) according to the manufacturer's instructions. PCR product was resolved on 5% non-denaturing polyacrylamide gel and stained in ethidium bromide prior to imaging on the Kodak Gel Logic Imaging System. The percentage of exon skipping was calculated as [exon exclusion band/ (exon inclusion band + exon exclusion band)] x 100 with a correction factor for the amount of ethidium bromide per base pair of DNA. See Supplementary Table S3 for primer sequences.

For quantitative RT-PCR, approximately 10 ng RNA was added to EXPRESS One-Step SuperScript qRT-PCR Universal (Life Technologies) with custom designed or predesigned Taqman primer and probe sets (see Supplementary Table S4 for sequences). All quantification was performed by the relative standard curve method and normalized to Ribogreen.

### Western blot

Total cellular protein was prepared by cell lysis in Radioimmunoprecipitation assay (RIPA) buffer (Sigma-Aldrich) containing ethylenediaminetetraacetic acid-free cOmplete Protease Inhibitor Cocktail Tablet (Roche). Cells were further lysed by passage through a needle five times, then cellular debris pelleted by refrigerated centrifugation. Protein concentration of the supernatant was determined by DC protein assay (Bio-Rad). 10–40 μg protein lysate was separated on a precast 4–20% Bis-Tris gel (Life Technologies) and transferred by iBlot (Life Technologies). See Supplementary Table S5 for antibody information.

### 5′ rapid amplification of cDNA ends

To identify endonuclease cleavage sites, 5′ rapid amplification of cDNA ends (5′ RACE) was performed using the First Choice RLM-RACE kit (Life Technologies) with minor modifications. Briefly, the 5′ RACE adapter was ligated to the free 5′ phosphate in 2 μg of RNA using T4 ligase. Reverse transcription was performed using SuperScript II followed by outer PCR for 35 cycles at 60°C annealing temperature. If necessary, a nested inner PCR was performed for an additional 12 cycles. 5′ RACE products were visualized on 2% agarose gels stained in ethidium bromide. To determine cleavage sites, bands were gel isolated, amplified by TOPO TA Cloning (Life Technologies), and sequenced (Retrogen). See Supplementary Table S3 for 5′ RACE primer sequences.

### Cellular fractionation

Cellular fractionation was performed as described in (17). Purity of each fraction was assessed by distribution of RNA (*STAT3* intron 21, nucleus; *RN7SL1*, cytoplasm) and protein (Histone H3, chromatin; U1A, nucleoplasm; α-tubulin, cytoplasm).

### RNA immunoprecipitation

5 × 10^6^ HeLa cells were transfected with 50 nM ASO using 3 μg/ml Cytofectin transfection reagent for 24 h. RNA immunoprecipitation was performed using the Magna RIP^TM^ RNA-Binding Protein Immunoprecipitation Kit (EMD Millipore) according to the manufacturer's instructions with 3 μg ChIPAb+ RNA Pol II monoclonal antibody (EMD Millipore) or 3 μg mouse IgG. Following RNA purification, residual DNA was removed by digestion with amplification grade DNase I (Life Technologies). Successful RNA polymerase II (RNAPII) immunoprecipitation was confirmed by western blot. RNA immunoprecipitation efficiency and specificity was assessed by calculating the percent input and fold enrichment for *GAPDH* (an RNAPII target) and *RN7SL1* (an RNAPIII target) (data not shown).

### ASO *in vivo* administration

For central nervous system administration, a single ASO dose of 500 μg in phosphate buffered saline (PBS) was delivered by intracerebroventricular injection to 8-week-old C57BL/6 female mice (JAX). Four weeks post-treatment, whole brain and thoracic spinal cord tissue was lysed using FastPrep Lysing Matrix Tubes (MP-Biomedicals) in RLT buffer (Qiagen) containing 1% beta-mercaptoethanol and RNA was isolated using the RNeasy kit (Qiagen). No body weight change or inflammation (Aif1 qRT-PCR) was observed.

For systemic administration, 6-week-old BALB/c male mice (JAX) were dosed twice per week for 3 weeks with 50 mg/kg ASO in PBS. Liver was harvested 48 h after the last dose. Tissue was lysed using FastPrep Lysing Matrix Tubes (MP-Biomedicals) in guanidine isothiocyanate (Life Technologies) containing 8% beta-mercaptoethanol and RNA was isolated using the RNeasy kit (Qiagen). No changes in body weight, organ weight or plasma markers were observed.

## RESULTS

### Uniformly modified 2′-MOE ASOs disrupt proper pre-mRNA processing and cause mRNA reduction *in vitro*

**Figure 1.**
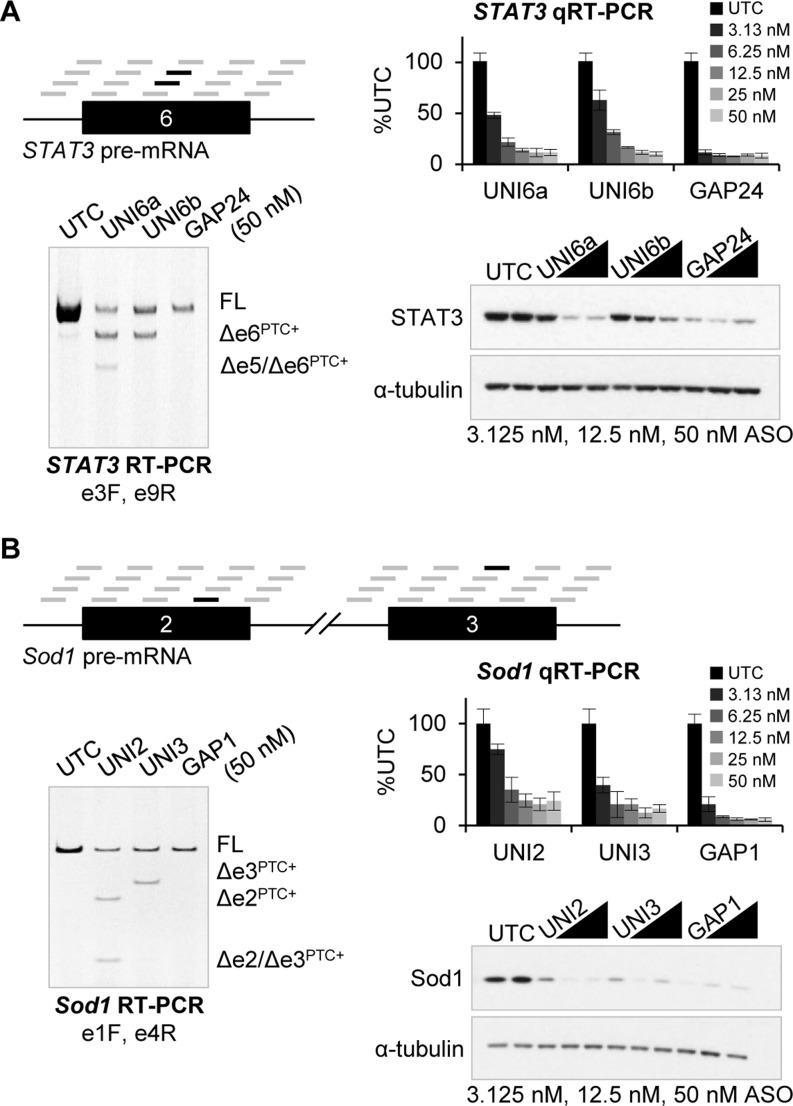
Uniform 2′-MOE ASOs alter mRNA processing and promote target reduction. (**A**) RNA and protein analysis from HeLa cells transfected with uniform 2′-MOE ASOs (UNI6a and UNI6b) or 2′-MOE gapmer ASO (GAP24) targeting *STAT3* for 24 h. Schematic depicts uniform 2′-MOE ASOs screened across *STAT3* exon 6 and the surrounding splice junctions. The most active ASOs selected for further studies are shown in black. Pre-mRNA splicing was analyzed by RT-PCR using primers in exon 3 and exon 9 and visualized by separation on an ethidium bromide stained polyacrylamide gel. Bands were confirmed by sequencing. RNA and protein expression were measured by qRT-PCR with a Taqman primer and probe set located in exon 3 and by western blot, respectively. (**B**) *Sod1* expression from bEnd.3 cells transfected with uniform 2′-MOE ASOs (UNI2 and UNI3) and 2′-MOE gapmer ASO (GAP1) targeting *Sod1* for 24 h. Pre-mRNA splicing was analyzed by RT-PCR using primers in exon 1 and exon 4. RNA and protein expression was measured by qRT-PCR with a Taqman primer and probe set located in exon 1 and by western blot, respectively. Bar graph is mean ± SD (*n* = 3).

We performed an unbiased screen of 18-mer uniformly modified 2′-MOE ASOs with phosphorothioate backbone. Uniformly modified 2′-MOE ASOs act through steric hindrance and do not support RNase H or RISC activity. The ASOs were spaced every three nucleotides across *STAT3* exon 6 and *Sod1* exon 2 and exon 3 including the 5′ and 3′ splice sites to identify ASOs that redirected splicing. Human cervical epithelial cells (HeLa) and mouse brain endothelial cells (bEnd.3) were used for all human *STAT3* and mouse *Sod1* experiments, respectively. Cells were transfected with 50 nM uniform 2′-MOE ASOs and 24 h post-transfection exon skipping was analyzed by RT-PCR. In each case, the uniform 2′-MOE ASOs with the strongest effect on splicing had binding sites located centrally in the exon (Figure 1). Although there are no characterized exonic splicing enhancer elements in *STAT3* exon 6 or *Sod1* exon 2 or exon 3, ESEfinder 3.0 (18) sequence analysis showed the active uniform 2′-MOE ASOs blocked putative exonic splicing enhancers containing consensus motifs for serine/arginine-rich proteins (Supplementary Figure S1). Uniform 2′-MOE ASOs with binding sites in *STAT3* exon 6 (UNI6a and UNI6b) and *Sod1* exon 2 (UNI2) and exon 3 (UNI3) caused decreased expression of the full-length transcript and appearance of a lower molecular weight band, indicative of exon skipping (Figure 1). The identity of the lower molecular weight product was confirmed by sequencing. Two of the uniform 2′-MOE ASOs also caused double exon skipping—UNI6a caused double skipping of *STAT3* exon 5 and exon 6 and UNI2 caused double skipping of *Sod1* exon 2 and exon 3. Sequence analyses of the open reading frames shows that single and double exon skipping creates PTC-containing mRNAs. As a control, cells were transfected with previously identified 2′-MOE gapmer ASOs (20-mers with five 2′-MOE substitutions on the 5′ and 3′ wings) targeting *STAT3* (GAP24) or *Sod1* (GAP1) that function through RNase H to reduce the target RNA (Figure 1). The 2′-MOE gapmer ASOs were more potent than the uniform 2′-MOE ASOs at downregulating the targets.

Unlike the targeting of constitutive exons in *STAT3* and *Sod1* mRNA, we also evaluated ASOs designed to promote skipping of an alternative exon in human *HNRNPH1* that contains exonic ultraconserved elements associated with alternative splicing-NMD (19). HeLa cells treated with uniform 2′-MOE ASOs targeting *HNRNPH1* alternative exon 4 (UNI4a and UNI4b) showed decreased expression of full-length *HNRNPH1* and increased exon 4 skipping thereby enhancing the amount of PTC-containing mRNA present in the cell (Supplementary Figure S2A).

We determined if uniform 2′-MOE ASO treatment resulted in *STAT3*, *Sod1* and *HNRNPH1* mRNA reduction by real-time RT-PCR (qRT-PCR). Similar to RNase H-dependent 2′-MOE gapmer ASOs, treatment with uniform 2′-MOE ASOs resulted in robust, dose-dependent RNA knockdown with maximum reductions of 70–85% compared to untransfected control cells. Correspondingly, dose-dependent reductions in protein levels were also observed (Figure 1, Supplementary Figure S2A). Decreased target RNA expression was observed 8 h post-transfection of ASO, with maximum reduction at 24 h and effects lasting 72 h (Supplementary Figure S3).

To determine if additional ASO chemical modifications supported RNA reduction, we designed sequence-matched ASOs to the active uniform 2′-MOE ASO binding sites and tested their ability to decrease the target mRNA. ASOs of multiple chemistries including uniform 2′-*O*-methyl, 2′,4′-constrained ethyl mixmers, and uniform 2′-MOE gapmers resulted in mRNA reduction (Supplementary Figure S4). Uniform 2′-*O*-methyl ASOs were consistently the least effective, and in general the 2′,4′-constrained ethyl mixmers yielded mRNA reductions similar to uniform 2′-MOE ASOs. Sequence-matched uniform 2′-MOE and 2′-MOE gapmer ASOs targeting two different sites on *STAT3* were nearly equipotent (IC_50_ values of 2′-MOE gapmer ASOs were 1.1–1.2 times higher). For *Sod1* and *HNRNPH1*, the 2′-MOE gapmers had IC_50_ values 1.5–23 times higher than sequence-matched uniform 2′-MOE ASOs (Supplementary Figure S4).

While 2′-MOE gapmer ASOs are known to cause RNA reduction through an RNase H enzymatic cleavage event, the molecular pathway by which these steric blocking ASOs decrease mRNA expression has not been previously characterized. We hypothesize that by redirecting splicing to generate PTC-containing mRNAs, the ASOs may invoke the NMD pathway to degrade the misprocessed transcripts. Because the PTCs are located more than 55 nucleotides upstream of the last exon–exon junction in each transcript, the messages are predicted NMD substrates but this can only be verified experimentally.

### Uniform 2′-MOE ASOs direct transcripts to the nonsense-mediated mRNA decay pathway for degradation

**Figure 2.**
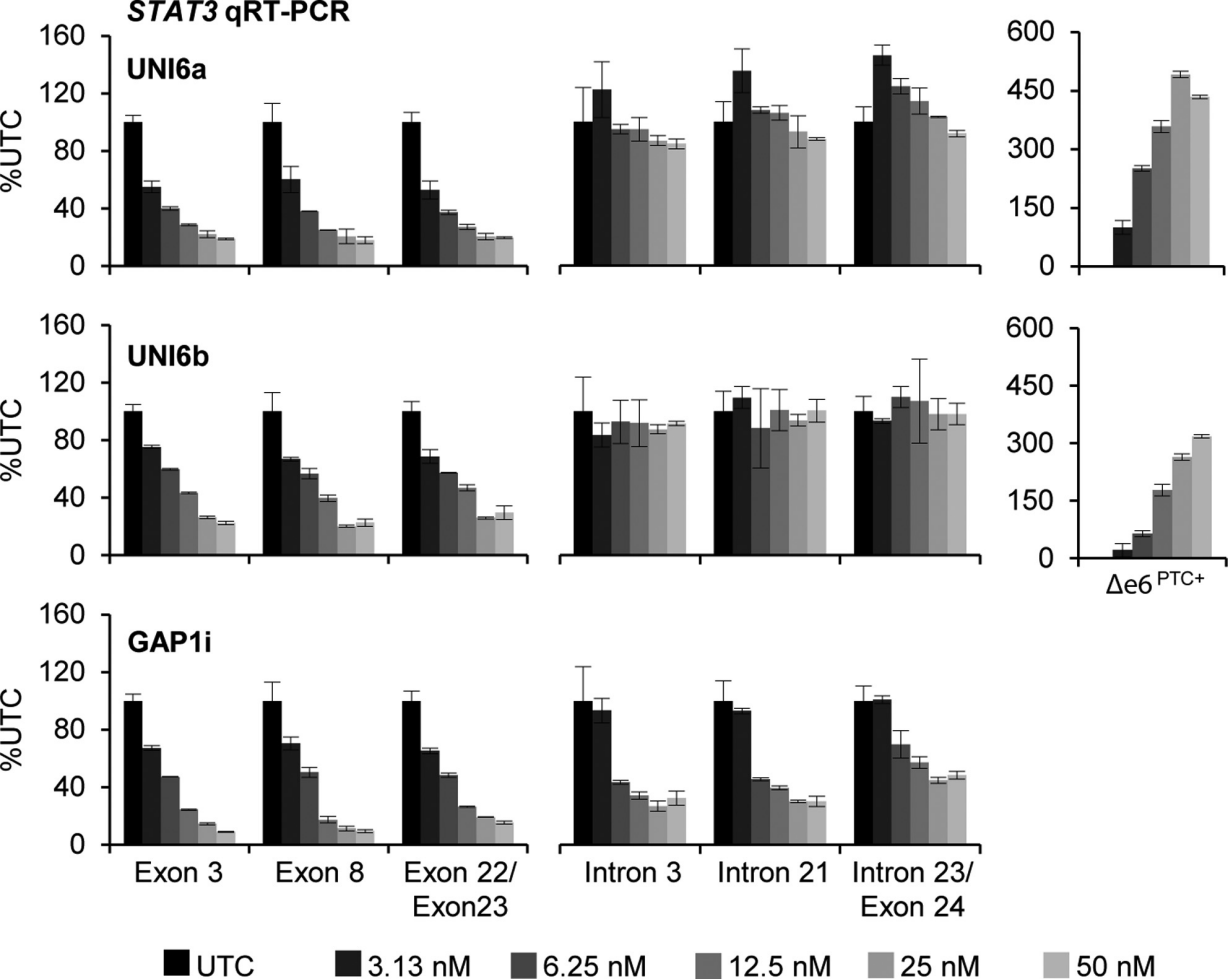
Uniform 2′-MOE ASOs targeting human *STAT3* exon 6 reduce *STAT3* mRNA after splicing has occurred. Following 24 h treatment with increasing dose of UNI6a and UNI6b ASO, qRT-PCR was performed at the indicated regions across the pre-mRNA and mRNA of *STAT3*, including a Taqman primer and probe set specific for the novel exon–exon junction created by exon 6 skipping. As a positive control for pre-mRNA reduction, cells were treated with a 2′-MOE gapmer ASO (GAP1i) targeting *STAT3* intron 1. Mean ± absolute deviation (*n* = 2).

To determine if uniform 2′-MOE ASOs reduced the level of pre-mRNA or mRNA, we treated cells with UNI6a and UNI6b and analyzed *STAT3* pre-mRNA and mRNA expression by qRT-PCR. Uniform 2′-MOE ASO treatment resulted in dose-dependent reduction of *STAT3* mRNA upstream and downstream of the ASO binding site. In contrast, no reductions were observed in pre-mRNA levels including the last intron–exon junction in the transcript (Figure 2). A 2′-MOE gapmer ASO targeting *STAT3* intron 1 (GAP1i) served as a positive control for pre-mRNA knockdown by RNase H and showed dose-dependent reductions in all regions of the transcript. This data suggest that uniform 2′-MOE ASOs act to decrease the expression of *STAT3* after splicing has occurred, consistent with an NMD process.

**Figure 3.**
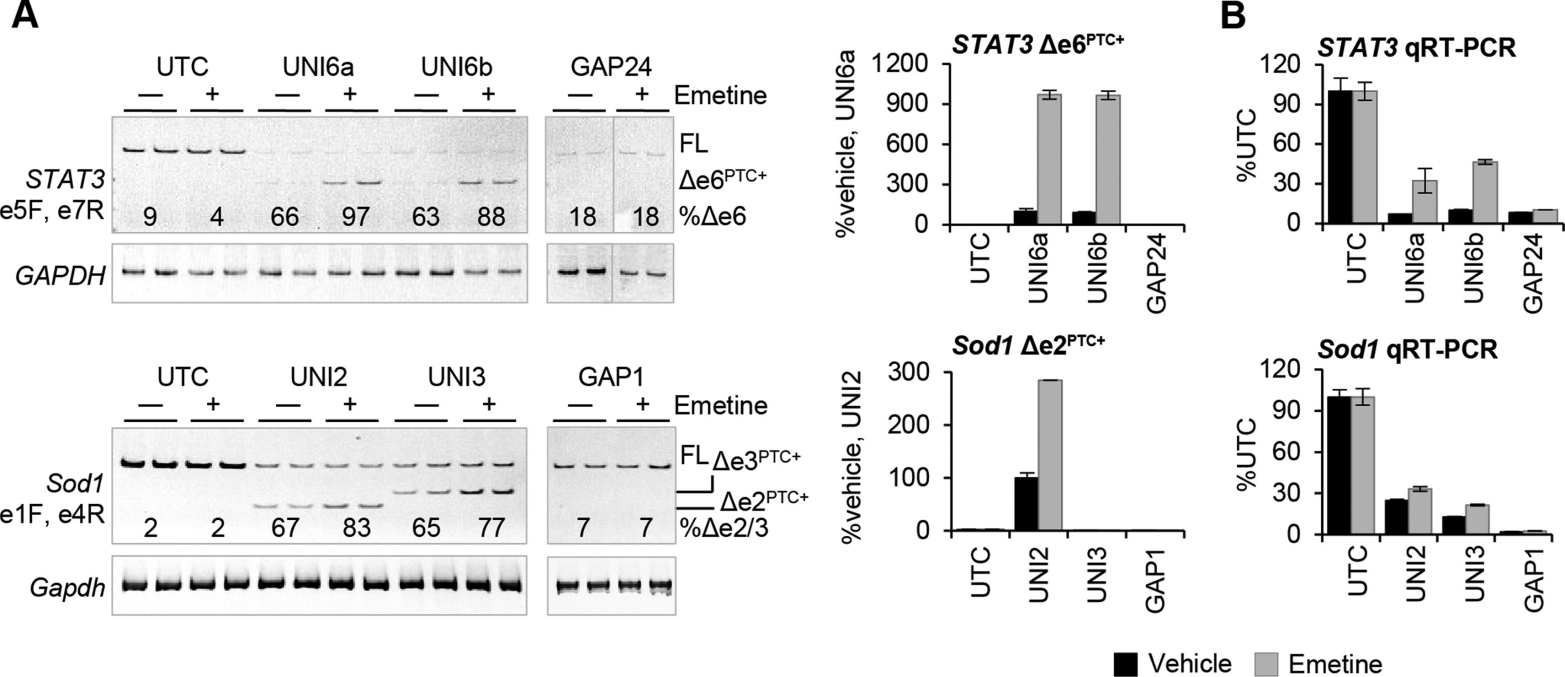
Uniform 2′-MOE ASOs cause mRNA reduction through a translation-dependent decay process. (**A**) Cells were transfected with 50 nM uniform 2′-MOE or 2′-MOE gapmer ASO for 24 h. Translation was inhibited by emetine addition the last 4 h of ASO treatment. A polyacrylamide gel was used to visualize RT-PCR results for *STAT3* and *Sod1* pre-mRNA splicing. Band intensity was quantified to calculate the percent exon skipping in the absence and presence of emetine. Stabilization of the PTC-containing transcripts was analyzed by qRT-PCR using Taqman primer and probe sets specific for the novel exon–exon junctions generated by exon skipping. (**B**) Total RNA levels were quantified by qRT-PCR using Taqman primer and probe sets in *STAT3* exon 3 and *Sod1* exon 1. Bar graph is mean ± absolute deviation (*n* = 2).

Translation is required for NMD and, therefore, translation inhibition indirectly blocks NMD as well as several other translation-dependent decay processes in the cell (20). We inhibited translation following ASO treatment to determine if uniform 2′-MOE ASOs caused mRNA reduction through a translation-dependent decay pathway. Translation inhibition by emetine stabilized the *STAT3*, *Sod1* and *HNRNPH1* PTC-containing transcripts 3- to 9-fold (Figure 3A and Supplementary Figure S2B). The level of stabilization was quantified by qRT-PCR using Taqman primer and probe sets specific to the novel exon–exon junctions generated by alternative splicing. Translation inhibition also attenuated the ability of the uniform 2′-MOE ASOs to decrease the expression of the target mRNA suggesting the ASOs act to promote target reduction, at least in part, through a translation-dependent decay process (Figure 3B). In all cases, translation inhibition had no effect on RNA reduction by 2′-MOE gapmer ASOs consistent with the RNase H mechanism (Figure 3 and Supplementary Figure S2B).

**Figure 4.**
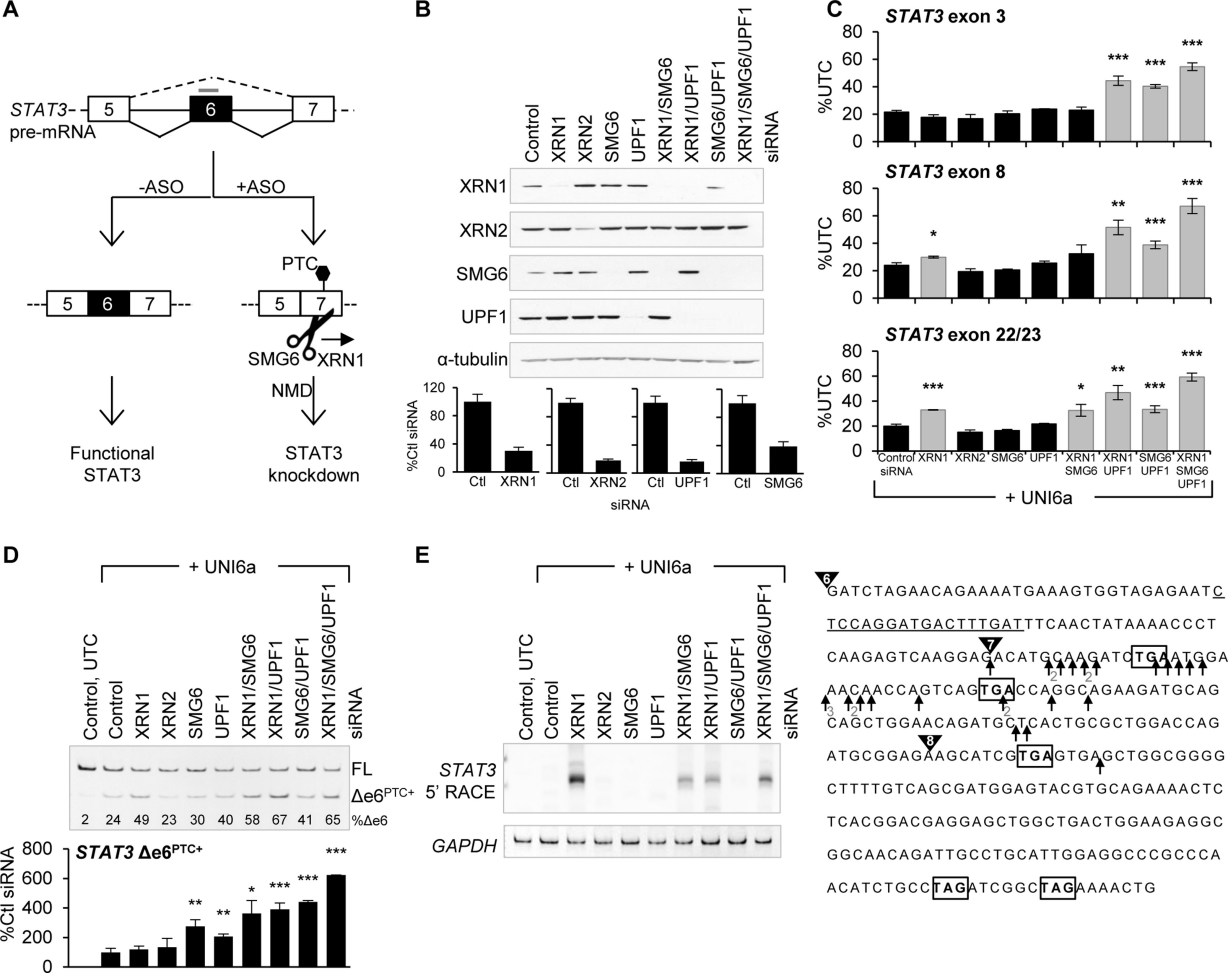
Uniform 2′-MOE ASOs direct aberrant *STAT3* mRNA to NMD. (**A**) Schematic diagram illustrating the effect of ASO action on *STAT3* expression. (**B**) HeLa cells were transfected with the indicated siRNAs for 48 h. Knockdown was assessed by western blot and qRT-PCR. (**C**) Following siRNA treatment, cells were transfected with UNI6a ASO for 24 h. *STAT3* RNA expression was analyzed by qRT-PCR with Taqman primer and probe sets upstream (exon 3) and downstream (exon 8 and exon 22/23) of the ASO binding site. (**D**) Effect of siRNA treatment on the expression level of the PTC-containing mRNA was analyzed by standard RT-PCR and qRT-PCR. (**E**) Agarose gel visualizing 5′ RACE products after siRNA and ASO treatment. Right panel: sequence of *STAT3* exons 6, 7 and 8 containing the UNI6a ASO binding site (underlined) and location of PTCs (boxed) upon exon 6 skipping. The arrows indicate the 5′ ends as determined by sequencing of individual clones from 5′ RACE. A total of 28 5′ RACE clones were sequenced. The numbers below the arrows indicate the number of clones sequenced with that cleavage site. Mean ± SD (*n* = 3); *P < 0.05, **P < 0.01, ***P < 0.005 determined by Student's t-test compared to control siRNA treatment.

To corroborate the data supporting NMD as the terminating mechanism of uniform 2′-MOE ASOs, we performed a detailed molecular characterization of NMD by inhibiting critical components of the pathway. Cells were transfected with siRNA to inhibit expression of the NMD factors UPF1 and SMG6 as well as the 5′-to-3′ exonucleases that function in the cytoplasm (XRN1) and nucleus (XRN2). In each case, the siRNAs promoted greater than 60% reduction in mRNA and protein (Figure 4B). NMD inhibition by combined treatment of UPF1 and SMG6 siRNA significantly impaired UNI6a-mediated reduction of *STAT3*, indicating the uniform 2′-MOE ASO reduces *STAT3* in an NMD-dependent manner (Figure 4C). XRN1 inhibition did not have any effect on the expression level of *STAT3* upstream of the ASO binding site location (shown by qRT-PCR for *STAT3* exon 3), but a different result was observed when the expression level was analyzed using *STAT3* qRT-PCR primers downstream of the ASO binding site (*STAT3* exon 8 and exon 22/23) (Figure 4C). In these assays, XRN1 inhibition attenuated STAT3 reduction by the ASO. These findings suggest XRN1 loads at an internal site and degrades *STAT3* 5′-to-3′ in the cytoplasm. Nuclear XRN2 inhibition had no effect on the activity of UNI6a, consistent with NMD as a cytoplasmic process (Figure 4C). The most effective condition to block ASO activity was combined UPF1, SMG6 and XRN1 siRNA treatment. These conditions attenuated the reduction of *STAT3* by more than 30% (Figure 4C) and stabilized the PTC-containing mRNA 6-fold (Figure 4D) compared to control siRNA transfected cells.

We next examined if uniform 2′-MOE ASO treatment resulted in internal *STAT3* cleavage by performing 5′ RACE on RNA from cells transfected with UNI6a. We were unable to detect 5′ RACE products in cells treated with ASO alone, suggesting either the transcript is not cleaved or the cleaved transcript is rapidly degraded by cellular nucleases (Figure 4E). In support of the latter hypothesis, pretreatment with XRN1 siRNA allowed detection of internally cleaved *STAT3* transcripts with heterogeneous 5′ ends near the PTCs (Figure 4E). This cleavage pattern is consistent with documented SMG6 cleavage sites (21). Combined transfection of XRN1 and SMG6 siRNA decreased cleavage as evidenced by the reduced 5′ RACE band intensity compared to XRN1 siRNA alone (Figure 4E). Similar results were obtained by inhibition of UPF1, which recruits SMG6 to the NMD substrate (22).

The same detailed molecular experiments were also performed using uniform 2′-MOE ASOs targeting *Sod1* with similar results (Supplementary Figure S5).

### Uniform 2′-MOE ASOs reduce chromatin-associated *STAT3* RNA

**Figure 5.**
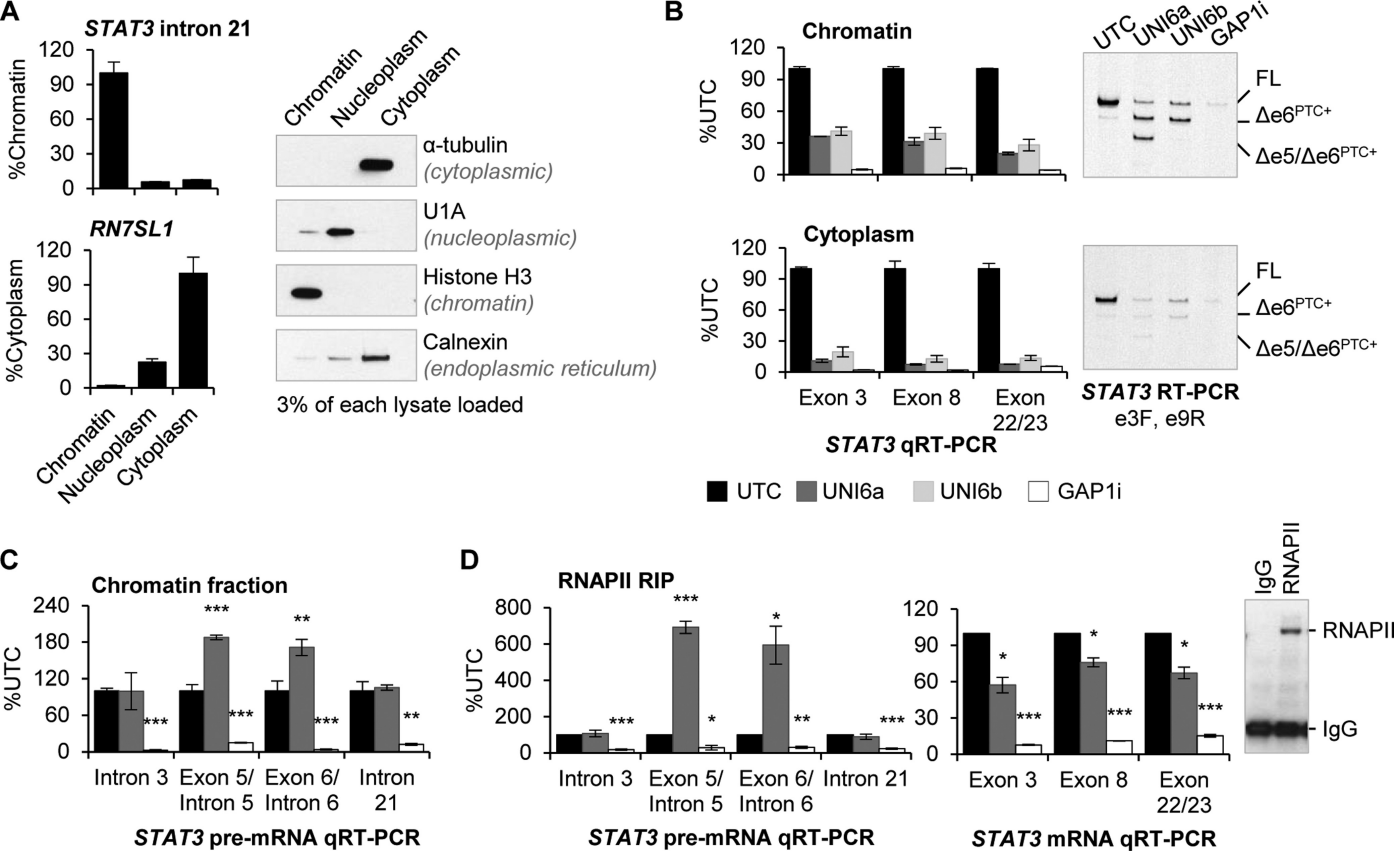
Uniform 2′-MOE ASOs cause intron retention and decreased levels of *STAT3* mRNA in association with the chromatin. (**A**) Purity of the cell fractions was assessed by qRT-PCR for a chromatin RNA marker (*STAT3* intron 21) and a cytoplasmic RNA marker (*RN7SL1*) as well as by western blotting for proteins enriched in the indicated compartments. (**B**) HeLa cells were transfected with uniform 2′-MOE (UNI6a and UNI6b) or 2′-MOE gapmer ASO (GAP1i) for 24 h, followed by cell fractionation. *STAT3* expression in each fraction was analyzed by qRT-PCR of the indicated regions of *STAT3* mRNA. Expression of improperly spliced mRNA was visualized by polyacrylamide separation of *STAT3* RT-PCR products. (**C**) *STAT3* pre-mRNA levels were analyzed in the chromatin fraction by qRT-PCR. (**D**) Following RNAPII immunoprecipitation, successful pull-down was shown by western blot (right panel) and RNAPII-bound *STAT3* was analyzed by qRT-PCR. Mean ± absolute deviation (*n* = 2); *P < 0.05, **P < 0.01, ***P < 0.005 determined by Student's t-test compared to untransfected control cells.

Despite strong inhibition of NMD by near complete knockdown of UPF1 and SMG6, uniform 2′-MOE ASOs still decreased *STAT3* RNA expression by about 40% compared to untransfected control cells. *STAT3* may be degraded by a cellular surveillance pathway prior to nuclear export, so we analyzed *STAT3* RNA expression in three cellular fractions—chromatin, nucleoplasm and cytoplasm. The purity of each fraction was carefully assessed by RNA and protein distribution. *STAT3* intron expression was enriched 15-fold and the cytoplasmic RNA *RN7SL1* was enriched 50-fold in the chromatin and cytoplasm, respectively (Figure 5A). Protein analysis showed strong enrichment of α-tubulin (cytoplasm), U1A (nucleoplasm) and histone H3 (chromatin) in the expected fractions. An endoplasmic reticulum marker, calnexin, was predominantly localized in the cytoplasm with weak nucleoplasm signal, indicating the nucleoplasm fraction contains the outer nuclear membrane (Figure 5A). The expected distribution of XRN1 (cytoplasm) and XRN2 (nucleus) was also observed (Supplementary Figure S6). Quantitative western blot for α-tubulin revealed the chromatin fraction contained less than 1% cytoplasmic contamination (Supplementary Figure S6).

Cells were transfected with UNI6a and UNI6b uniform 2′-MOE ASOs and STAT3 expression was analyzed in each cell fraction by qRT-PCR. Strikingly, we observed 60% reduction of *STAT3* in the chromatin fraction (Figure 5B). Additional *STAT3* reduction occurred in the cytoplasmic fraction, likely accounted for by NMD due to the decreased levels of the PTC-containing mRNA (Figure 5B). Pre-mRNA levels were not altered but uniform 2′-MOE ASO treatment impaired splicing of *STAT3* intron 5 and intron 6 resulting in intron retention at the exon–intron junctions closest to the ASO binding site (Figure 5C). Because these changes occurred in association with the chromatin, we interrogated the expression level and exon–intron architecture of *STAT3* RNA bound to RNAPII.

RNAPII was immunoprecipitated from untreated or UNI6a-treated HeLa cells. Successful pull-down was confirmed by western blot (Figure 5D). Pull-down with IgG antibody was used as a negative control. Following immunoprecipitation, RNAPII-bound RNA was purified. UNI6a treatment resulted in a 6-fold increase in *STAT3* intron 5 and intron 6 retention while pre-mRNA levels further upstream or downstream of the ASO binding site remained unaffected. In addition, RNAPII-bound *STAT3* mRNA was significantly reduced (Figure 5D). Cumulatively, this data suggest that in addition to the anticipated RNA reduction by exon skipping-NMD, uniform 2′-MOE ASOs targeting *STAT3* also promoted intron retention and activation of nuclear RNA surveillance pathways for decay.

### Uniform 2′-MOE ASOs disrupt proper mRNA processing and cause mRNA reduction *in vivo*

**Figure 6.**
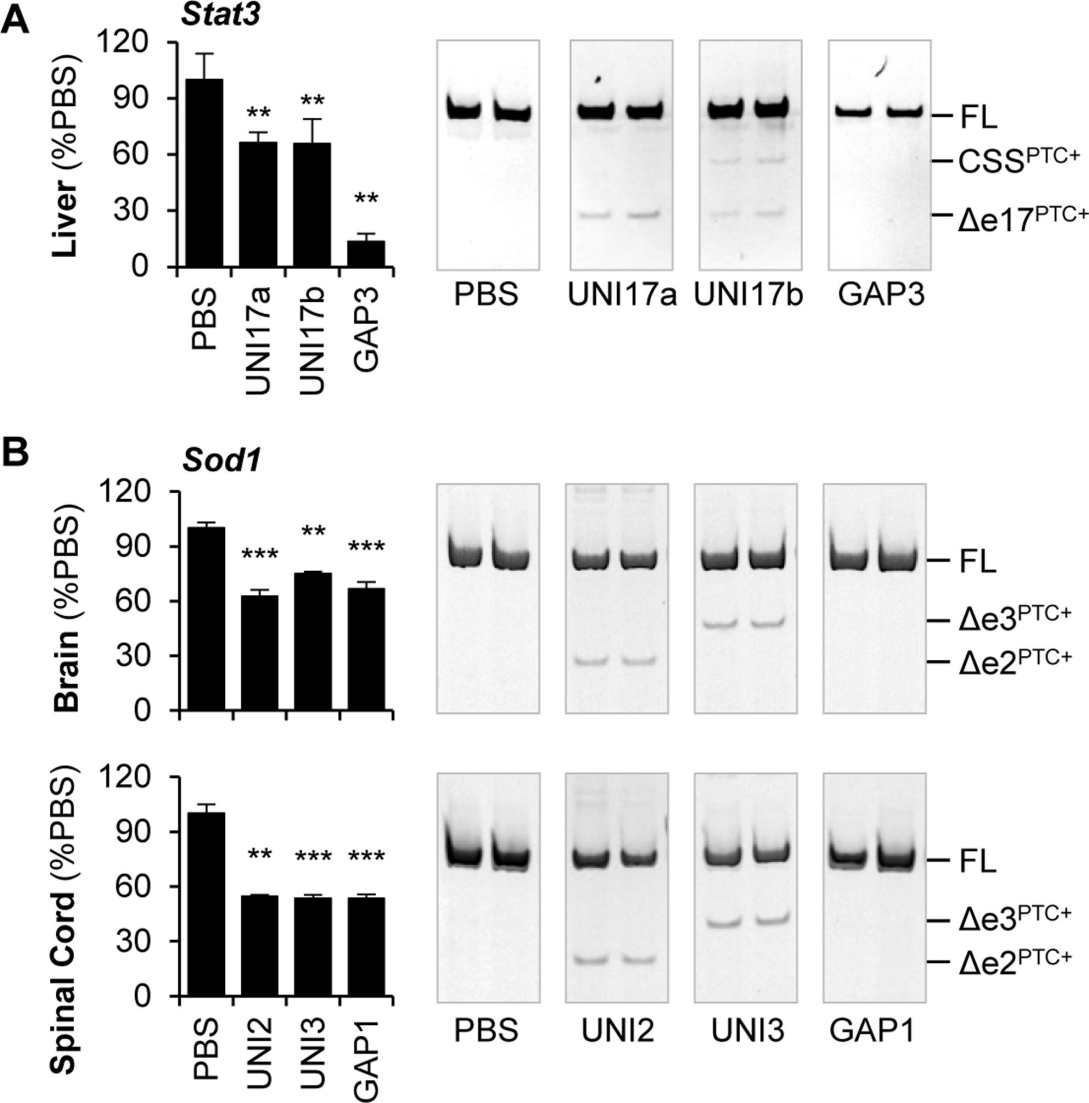
Uniform 2′-MOE ASOs cause reduction of target RNA *in vivo*. (**A**) Systemic delivery of uniform 2′-MOE ASOs targeting mouse *Stat3* exon 17 (UNI17a and UNI17b) causes exon 17 skipping and utilization of a cryptic 5′ splice site (CSS) shown by RT-PCR and *Stat3* knockdown in the liver shown by qRT-PCR. A 2′-MOE gapmer ASO (GAP3) is used as a positive control for *in vivo* knockdown. (**B**) Following central delivery of uniform 2′-MOE ASOs targeting mouse *Sod1* (UNI2 and UNI3), brain and spinal cord tissue was analyzed for exon skipping by RT-PCR and *Sod1* knockdown by qRT-PCR. Positive control 2′-MOE gapmer ASO (GAP1) is shown. Mean ± SEM (*n* = 4 per group); **P < 0.01, ***P < 0.005 determined by Student's t-test compared to PBS treated mice.

STAT3 is overactive in a variety of cancers, so STAT3 inhibition may be a viable approach for cancer therapeutics (23). To determine if steric blocking ASOs could reduce *Stat3* RNA *in vivo*, we designed uniform 2′-MOE ASOs complementary to mouse *Stat3* exon 6 (UNI6c and UNI6d) and exon 17 (UNI17a and UNI17b) and first confirmed they acted through splicing redirected-NMD *in vitro*. Treatment of bEnd.3 cells with UNI6 and UNI17 ASOs resulted in skipping of exon 6 and exon 17, respectively, and decreased *Stat3* expression (Supplementary Figure S7). Both exon 6 and exon 17 skipped transcripts contain PTCs. RT-PCR on RNA isolated from UNI17b treated cells revealed an additional lower molecular weight band that was confirmed by sequencing to be partial exon 17 skipping with utilization of a cryptic 5′ splice site. Translation inhibition by emetine treatment greatly stabilized the PTC-containing transcripts and significantly impaired the ability of the uniform 2′-MOE ASOs to knock down *Stat3* (Supplementary Figure S7). These data show that uniform 2′-MOE ASOs cause reduction of mouse *Stat3* through a similar terminating mechanism as human *STAT3*. For *in vivo* experiments, uniform 2′-MOE ASOs (UNI17a and UNI17b) and a 2′-MOE gapmer ASO (GAP19) targeting mouse *Stat3* were administered systemically by subcutaneous injection. Uniform 2′-MOE ASOs resulted in weak exon 17 skipping and a significant 30–35% decrease in *Stat3* expression in the liver, but were much less efficacious than the 2′-MOE gapmer ASO (Figure 6A). In this case, the RNase H mechanism provides a stronger *in vivo* knockdown than the terminating mechanism of splicing redirected-NMD.

Mutations in SOD1 are causative for familiar amyotrophic lateral sclerosis, a neurodegenerative disease, due to a toxic gain of function of the mutant protein in the central nervous system (24). To determine if steric blocking ASOs could reduce *Sod1* in the central nervous system, UNI2 and UNI3 uniform 2′-MOE ASOs targeting mouse *Sod1* were administered by intracerebroventricular injection. The uniform 2′-MOE ASOs caused weak skipping of *Sod1* exon 2 and exon 3 in the brain and spinal cord, and *Sod1* levels were significantly reduced 25–50%, which is similar to the level achieved with the same dose of an RNase H-dependent 2′-MOE gapmer ASO (Figure 6B).

## DISCUSSION

ASOs that function through RNase H and RISC take advantage of endogenous enzymes to degrade target RNA. Here, we show that steric blocking ASOs can exploit endogenous cellular surveillance pathways in the nucleus and cytoplasm to achieve RNA reduction. We rationally designed ASOs to redirect nuclear pre-mRNA splicing and promote formation of aberrant PTC-containing mRNAs. By doing so, we can direct these transcripts to NMD upon nuclear export. Uniform 2′-MOE ASOs act in the nucleus to redirect splicing and trigger decay, in part, through a cytoplasmic translation-dependent process. In contrast, 2′-MOE gapmer ASOs act in the nucleus and are capable of reducing pre-mRNA in a translation-independent manner. NMD inhibition by emetine treatment and by siRNA knockdown of critical NMD factors stabilized the PTC-containing transcripts and partially prevented ASO-mediated target reduction. We found that PTC-containing mRNAs generated by ASO action were recognized by UPF1 and subsequently cleaved by SMG6. The cleaved transcripts were then degraded by XRN1 resulting in target knockdown in the cytoplasm. Therefore, while 2′-MOE gapmer ASOs cause target cleavage through direct recruitment of the RNase H enzyme, uniform 2′-MOE ASOs indirectly recruit SMG6 to cleave the target mRNA.

We also sought to compare the potencies of ASOs that act through NMD-dependent and RNase H-dependent mechanisms. When comparing the ability of the ASOs to cause target reduction, the uniform 2′-MOE ASOs are typically less potent than 2′-MOE gapmer ASOs. In a few cases, the uniform 2′-MOE ASOs were equipotent to the 2′-MOE gapmer ASOs, including *in vitro* transfection of sequence-matched ASOs to two sites on *STAT3* (Supplementary Figure S4) and *in vivo* delivery of ASOs targeting independent sites in *Sod1* (Figure 6B). However, it is important to note that many factors including the depth of screening, ASO chemistry and target site accessibility can be determinants of ASO activity. Therefore, it is expected that upon further optimization, the activity of NMD-dependent and RNase H-dependent ASOs used in this study could be improved.

We observed that ASO treatment, unexpectedly, also reduced chromatin-associated *STAT3* mRNA expression prior to cytoplasmic decay by the NMD pathway. Reduced *STAT3* in the chromatin fraction was independently confirmed by a significant reduction in RNAPII-bound *STAT3* mRNA. Loss of chromatin-associated *STAT3* mRNA cannot be explained by impaired transcription elongation downstream of the targeted exon because chromatin-associated, or RNAPII-bound, pre-mRNA levels were not reduced by uniform 2′-MOE ASO treatment. These data suggest that *STAT3* mRNA is degraded after RNAPII has reached the 3′ end of the gene, while still associated with RNAPII on chromatin. Previously it was reported that intron retention does not affect RNAPII elongation kinetics (25,26), but impairs RNA release from the site of transcription (27,28). Retention of unprocessed transcripts is mediated by Rrp6, which imposes an RNA quality control checkpoint at the 3′ end of the gene (28,29). Xrn2 is also thought to participate in the elimination of unspliced transcripts that are produced during transcription (30). Our data showed that ASO-mediated intron retention and exon skipping had no effect on transcription elongation but resulted in chromatin- and RNAPII-associated decay, and is consistent with these reports. We hypothesize that *STAT3* intron retention and/or exon skipping results in impaired RNA release from the transcription site and subsequent nuclear decay by the exosome and Xrn2.

The finding that steric blocking ASOs can reduce the level of mRNA has been previously reported (13–15). However, our studies aimed at characterizing the terminating mechanism of ASOs that promote splicing redirected-NMD are noteworthy for continued development of ASOs as therapeutic agents. If splicing redirected-NMD is to be pursued as a therapeutic strategy, then the potential for ribosome read through of aberrant transcripts and expression of truncated and potentially toxic proteins must be carefully examined. The efficiency of NMD varies between tissues due to variable expression of the NMD factors (31) and contributes to how disease manifests in different tissues (32). If ASOs produce PTC-containing mRNAs in tissues with inefficient NMD, there may be a higher probability of translating these misprocessed transcripts into truncated proteins of unknown function.

Steric blocking ASOs are amenable to a wide range of chemical modifications because there is no need to maintain the structural and chemical requirements for RNase H or AGO2 activity. Future experiments should focus on leveraging the additional chemical space to further improve pharmacodynamic and pharmacokinetic properties, particularly for increasing distribution to tissues of interest, of steric blocking ASOs.

## SUPPLEMENTARY DATA

Supplementary Data are available at NAR online, including Supplementary Figures S1–S5 and Supplementary Tables S1–S6.

SUPPLEMENTARY DATA

SUPPLEMENTARY DATA
