# Supplementary material for: Nonsense-mediated decay as a terminating mechanism for antisense oligonucleotides
Source: Nucleic Acids Res. 2014 Apr 3;42(9):5871–9. doi: 10.1093/nar/gku184 (PMC4027159; doi:10.1093/nar/gku184)
Supplement: SUPPLEMENTARY DATA [file supp_42_9_5871__index.html]

Nonsense-mediated decay as a terminating mechanism for antisense oligonucleotides — Nonsense-mediated decay as a terminating mechanism for antisense oligonucleotides — SUPPLEMENTARY DATA 

# Nonsense-mediated decay as a terminating mechanism for antisense oligonucleotides

## SUPPLEMENTARY DATA

**Files in this Data Supplement:**

- SUPPLEMENTARY DATA
