# Supplementary material for: Nonsense-mediated decay as a terminating mechanism for antisense oligonucleotides
Source: Nucleic Acids Res. 2014 Apr 3;42(9):5871–9. doi: 10.1093/nar/gku184 (PMC4027159; doi:10.1093/nar/gku184)
Supplement: SUPPLEMENTARY DATA [file supp_gku184_nar-00113-y-2014-File008.pdf]

SUPPLEMENTARY DATA

Supplemental Figure Legends

Supplemental Figure 1. Putative exonic splicing enhancer (ESE) elements as indicated by the presence of binding motifs for serine/arginine-rich proteins using ESEfinder 3.0. X-axis contains the sequence of human *STAT3* exon 6, mouse *Sod1* exon 2, and mouse *Sod1* exon 3. The binding sites for active uniform 2'-MOE ASOs are indicated by the black box. Y-axis is the value of the ESE prediction scores above the threshold set by the ESEfinder 3.0 software.

Supplemental Figure 2. Uniform 2'-MOE ASOs targeting *HNRNPH1*, an endogenous NMD substrate, enhance knockdown through NMD. A) *HNRNPH1* expression from HeLa cells transfected for 24 hours with uniform 2'-MOE ASOs (UNI4a and UNI4b) or 2'-MOE gapmer ASO (GAP8). Pre-mRNA splicing was measured by RT-PCR using primers located in exon 3 and exon 5. Band identity was confirmed by sequencing. *HNRNPH1* knockdown at the RNA and protein level was determined by qRT-PCR and Western blot, respectively. B) HeLa cells were transfected with 50 nM ASO for 24 hours. Translation was inhibited the last 4 hours of treatment by addition of emetine. Stabilization of *HNRNPH1* exon 4 skipped transcripts was analyzed by RT-PCR. Band intensity was quantified to calculate the percent exon skipping. C) HeLa cells were treated with UPF1 and SMG6 siRNA (10 nM each) for 48 hours, followed by 24 hour ASO transfection (50 nM). Stabilization of *HNRNPH1* exon 4 skipped transcripts was analyzed by RT-PCR and qRT-PCR. Bar graph is mean ± s.d. (n=3).

Supplemental Figure 3. RNA expression by qRT-PCR at the indicated time points after ASO transfection comparing onset and duration of action between uniform 2'-MOE and 2'-MOE gapmer ASOs targeting *STAT3*, *Sod1*, and *HNRNPH1*. Mean ± absolute deviation (n=2).

Supplemental Figure 4. Knockdown achieved with chemically modified ASOs. Cells were transfected for 24 hours with sequence-matched, chemically modified ASOs generated to the sites of active uniform 2'-MOE ASOs. Multiple chemistry motifs supported the mechanism of knockdown of *STAT3*, *Sod1*, and *HNRNPH1*. IC_{50} values indicate the ASO concentration required for 50% knockdown of the target and were calculated from the dose response curves. IC_{50} values were not determined (ND) for ASOs that did not achieve 50% knockdown over the tested dose range. Mean ± absolute deviation (n=2).

Supplemental Figure 5. Uniform 2'-MOE ASOs direct aberrant *Sod1* mRNA to NMD. A) Schematic diagram illustrating the effect of ASO action on *Sod1* expression. B) bEnd.3 cells were transfected with the indicated siRNAs for 48 hours. Knockdown was assessed by Western blot and qRT-PCR. C) Following siRNA treatment for 48 hours, cells were transfected with UNI2 ASO for 24 hours. *Sod1* RNA expression was analyzed by qRT-PCR with Taqman primer and probe sets upstream (exon 1) and downstream (exon 3/4 and exon 5) of the ASO binding site. D) Effect of siRNA treatment on the expression level of the PTC-containing mRNA was analyzed by standard RT-PCR and qRT-PCR. E)

Agarose gel visualizing 5' RACE products after siRNA and ASO treatment. Right panel: Sequence of *Sod1* exon 2, 3, and 4 containing the UNI2 ASO binding site (underlined) and location of PTCs (boxed) upon exon 6 skipping. The arrows indicate the 5' ends as determined by sequencing of individual clones from 5' RACE. A total of 32 5' RACE clones were sequenced. The numbers below the arrows indicate the number of clones sequenced with that cleavage site. Mean ± s.d. (n=3); *p<0.05, **p<0.01, ***p<0.005 determined by Student's t-test compared to control siRNA treatment.

Supplemental Figure 6. Purity of subcellular fractions by Western blot. Left panel: UPF1, SMG6, XRN1, and XRN2 were localized to the expected compartments. Right panel: Chromatin lysate was spiked with different ratios of cytoplasmic lysate to determine percent cytoplasmic contamination in the chromatin fraction. Less than 1% contamination is present according to the α-tubulin Western blot.

Supplemental Figure 7. Uniform 2'-MOE ASOs targeting mouse *Stat3* exon 6 and exon 17 behave similar to ASOs targeting human *STAT3*. A) bEnd.3 cells were treated for 24 hours with uniform 2'-MOE ASOs targeting exon 6 (UNI6c and UNI6d) and exon 17 (UNI17a and UNI17b) or with 2'-MOE gapmer ASO (GAP19). The last 4 hours of treatment, emetine was added to inhibit translation. Mouse *Stat3* expression was analyzed by qRT-PCR. Mean ± absolute deviation. B) Mouse *Stat3* splicing was measured by standard RT-PCR using primers flanking exon 6 or exon 17. Sequencing confirmed the band identity, including a cryptic 5' splice site (CSS) that was utilized upon treatment with UNI17b ASO as illustrated in the schematic.

Supplemental Figure 1

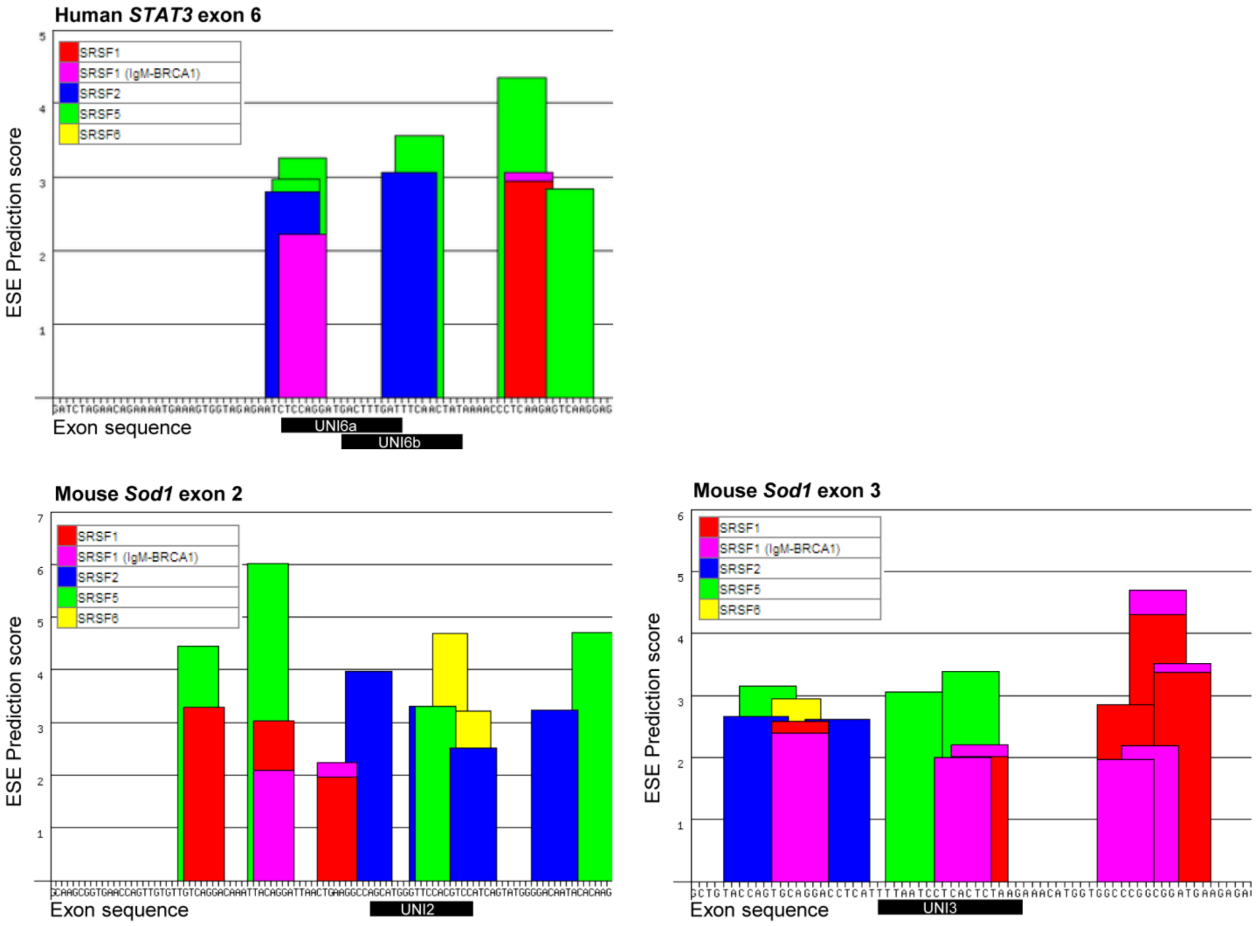

Supplemental Figure 2

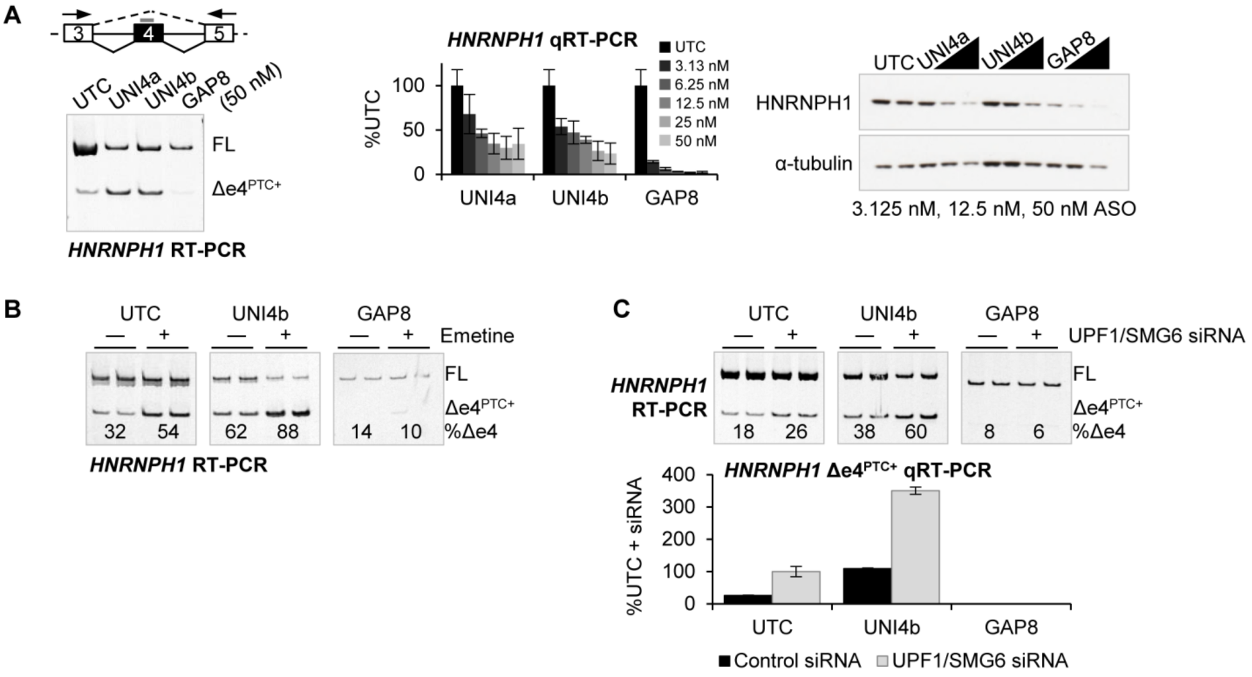

Supplemental Figure 3

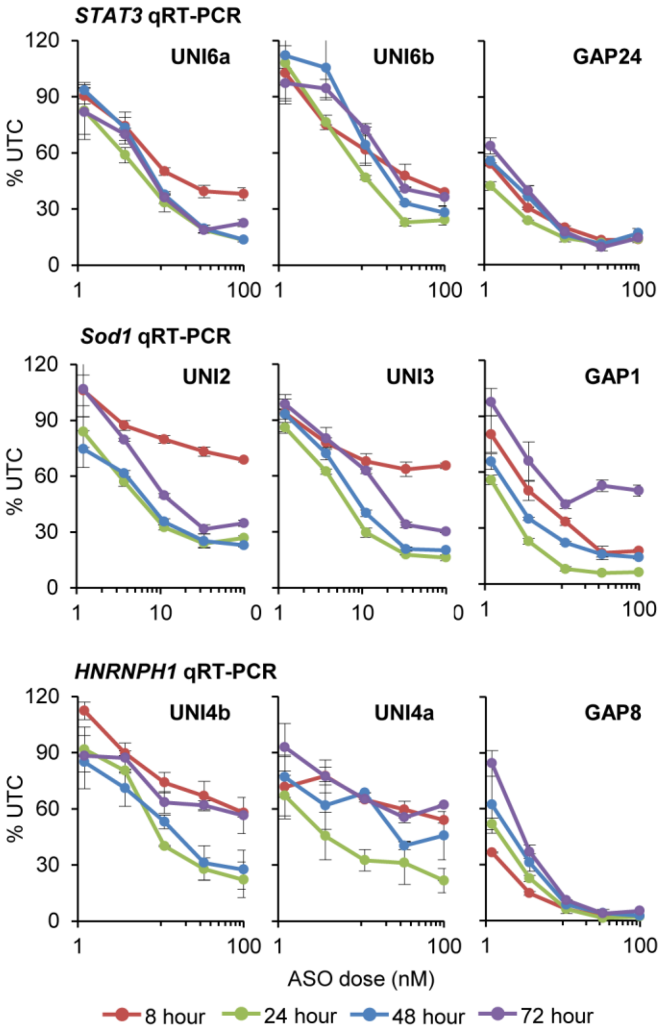

Supplemental Figure 4

```
Chemistry Notation (e=MOE, d=DNA, k=cEt, m=OMe)
               |-01 -02 -03 -04 -05 -06 -07 -08 -09 -10 -11 -12 -13 -14 -15 -16 -17 -18 -19 -20|
MOE            | eeeeeeeeeeeeeeeeee  |   (n=18)
GAP            |eeeeedddddddddddeeeee|   (n=20)
OME            | mmmmmmmmmmmmmmmmmm  |   (n=18)
DNA/cEt 1      | kkddkddkddkddkddkk  |   (n=18)
DNA/cEt 2      |   kddkddkddkddkddk  |   (n=16)
DNA/cEt 3      |kddkddkddkddkddk     |   (n=16)
MOE/cEt 1      | kkddkddkddkddkddkk  |   (n=18)
MOE/cEt 2      |   kddkddkddkddkddk  |   (n=16)
MOE/cEt 3      |kddkddkddkddkddk     |   (n=16)
```

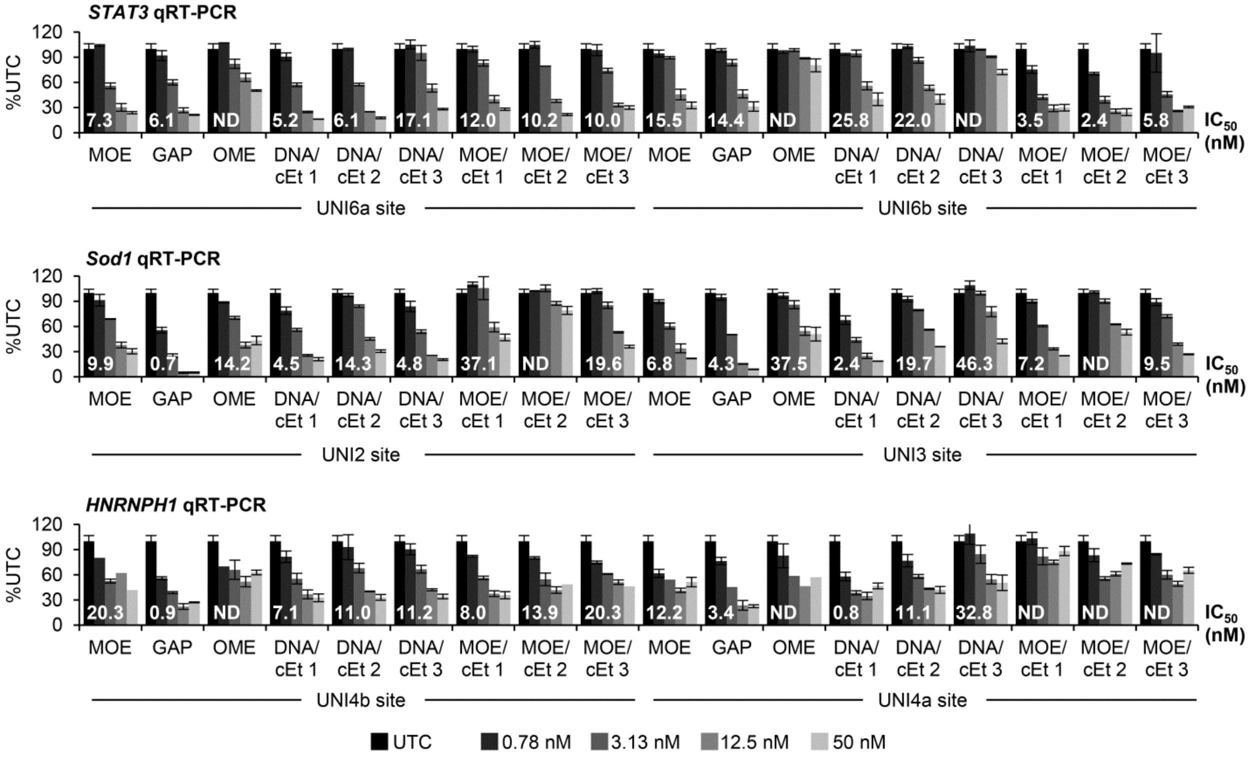

Supplemental Figure 5

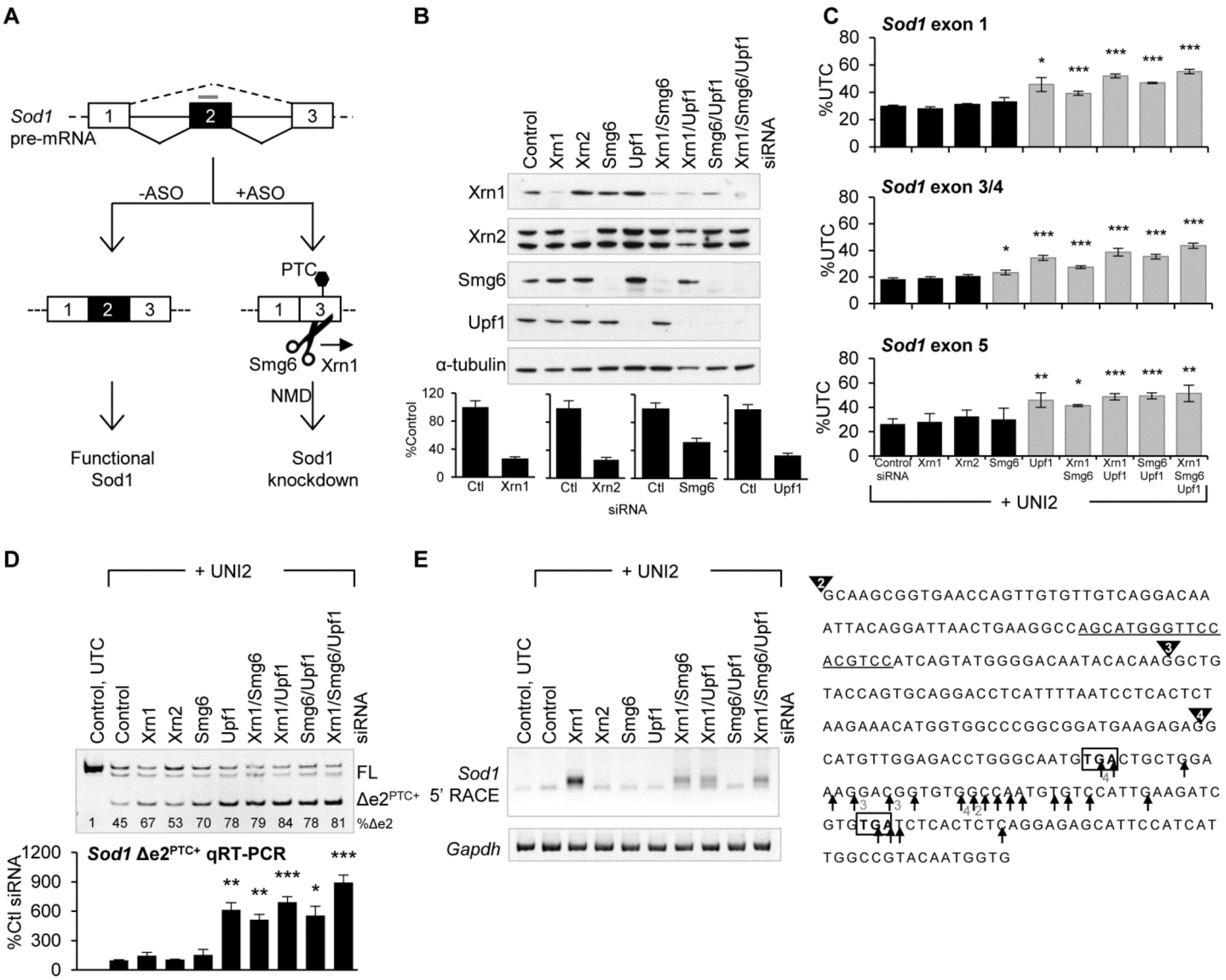

Supplemental Figure 6

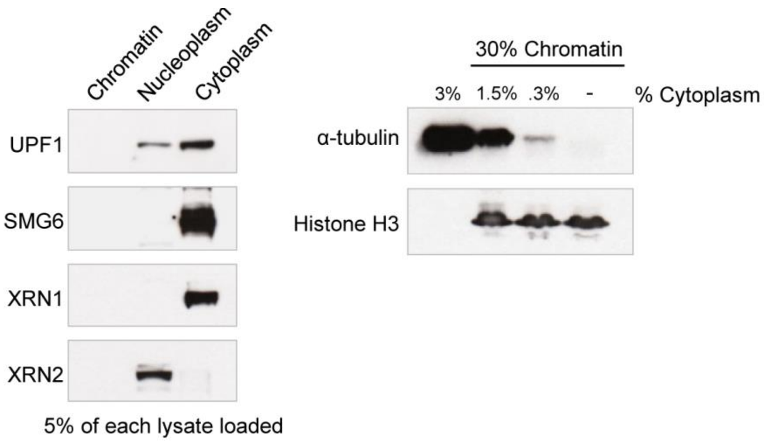

Supplemental Figure 7

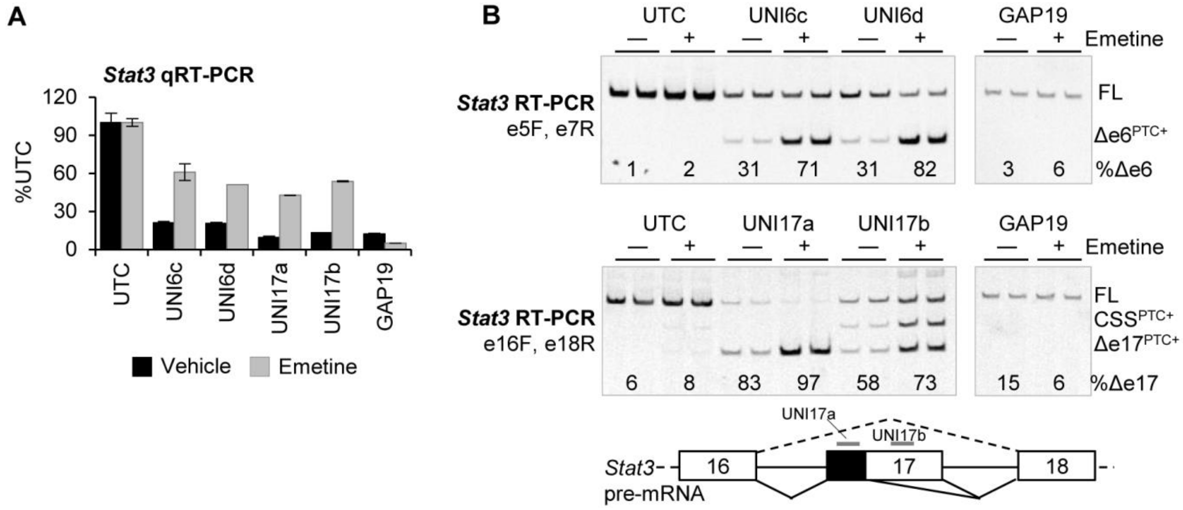

Supplemental Tables

Supplemental Table 1: ASO sequences

ASO	Target	Binding site	nt	Sequence	Chemistry
UNI6a	*STAT3* human	exon 6	18	ATCAAAGTCATCCTGGAG	Uniform 2'-MOE
UNI6b	*STAT3* human	exon 6	18	ATAGTTGAAATCAAAGTC	Uniform 2'-MOE
GAP1i	*STAT3* human	intron 1	20	TTTTGCATGATGTAACCACT	2'-MOE gapmer
GAP24	*STAT3* human	exon 24	20	CAGCAGATCAAGTCCAGGGA	2'-MOE gapmer
UNI6c	*Stat3* mouse	exon 6	18	AGTTGAAATCAAAGTCGT	Uniform 2'-MOE
UNI6d	*Stat3* mouse	exon 6	18	TCTTGAGGGTTTTGTAGT	Uniform 2'-MOE
UNI17a	*Stat3* mouse	exon 17	18	GGCTTAGTGAAGAAGTTC	Uniform 2'-MOE
UNI17b	*Stat3* mouse	exon 17	18	GTGGTGGACGAGAACTGC	Uniform 2'-MOE
GAP3	*Stat3* mouse	exon 3	20	GACTCTTGCAGGAATCGGCT	2'-MOE gapmer
GAP19	*Stat3* mouse	exon 19	20	GAAGCCCTTGCCAGCCATGT	2'-MOE gapmer
UNI2	*Sod1* mouse	exon 2	18	GGACGTGGAACCCATGCT	Uniform 2'-MOE
UNI3	*Sod1* mouse	exon 3	18	CTTAGAGTGAGGATTAAA	Uniform 2'-MOE
GAP1	*Sod1* mouse	exon 1	20	CCGTCGCCCTTCAGCACGCA	2'-MOE gapmer
UNI4a	*HNRNPH1* human	exon 4	18	CCACCGGCAATGTTATCC	Uniform 2'-MOE
UNI4b	*HNRNPH1* human	exon 4	18	AGCAAACTGCACGAAGGC	Uniform 2'-MOE
GAP8	*HNRNPH1* human	exon 8	20	AGCTTCACTGTTACATCCTA	2'-MOE gapmer

Supplemental Table 2: siRNA sequences

**Target**	**Sense Sequence**
XRN1, human	AGAUGAACUUACCGUAGAAdTdT
XRN2, human	GAGUACAGAUGAUCAUGUUdTdT
SMG6, human	GCUGCAGGUUACUUACAAGdTdT
UPF1, human	GAUGCAGUUCCGCUCCAUUdTdT
Xrn1, mouse	GAGGUGUUGUUUCGAAUUAdTdT
Xrn2, mouse	UUACAUAGCUGAUCGUUUAdTdT
Smg6, mouse	GAUCGGAACCUUCGUGUAAdTdT
Upf1, mouse	GAUGCAGUUCCGUUCCAUCdTdT

Supplemental Table 3: PCR primer sequences

**Target**	**Binding Site**	**Species**	**Sequence**
STAT3 forward	Exon 5	Human	TGGAGCAGCACCTTCAGGATGT
STAT3 reverse	Exon 7	Human	TCCAGCGCAGTGAGCATCTGTT
STAT3 forward	Exon 3	Human	AGTATAGCCGCTTCCTGCAAGAGT
STAT3 reverse	Exon 9	Human	CGGGTCTGAAGTTGAGATTCTGCT
Stat3 forward	Exon 5	Mouse	GATGTTGGAGCAGCATCTTCAGGA
Stat3 reverse	Exon 7	Mouse	TCCAGGGCTGTGAGCATCTGTT
Stat3 forward	Exon 16	Mouse	ACTCCTTGCCAGTTGTGGTGATCT
Stat3 reverse	Exon 18	Mouse	TTAGCCCATGTGATCTGACACCCT
Sod1 forward	Exon 1	Mouse	TGCAGGGAACCATCCACTTCGA
Sod1 reverse	Exon 4	Mouse	ACCGTCCTTTCCAGCAGTCACA
HNRNPH1 forward	Exon 3	Human	TCCAAATAGTCCTGACACGGCCAA
HNRNPH1 reverse	Exon 5	Human	TGGCCATAAGCTTTCGTGGTGGAT
GAPDH forward	Exon 6	Human, mouse	ACCACAGTCCATGCCATCAC
GAPDH reverse	Exon 7	Human, mouse	TCCACCACCCTGTTGCTGT
**5' RACE Primers**			
5' RACE Adapter			GCUGAUGGCGAUGAAUGAACACUGCGUU UGCUGGCUUUGAUGAAA
5' RACE Outer			GCTGATGGCGATGAATGAACACTG
5' RACE Inner			CGCGGATCCGAACACTGCGTTTGCTGGCT TTGATG
STAT3 RACE Outer reverse		Human	CAGGGAATTTGACCAGCAACCT
STAT3 RACE Inner reverse		Human	CGGGTCTGAAGTTGAGATTCTGCT
Sod1 RACE Outer reverse		Mouse	TGGTTTGAGGGTAGCAGATGAG

Supplemental Table 4: qRT-PCR primer sequences

**Target**	**Binding Site Location**	**Forward Sequence**<br>**Probe Sequence**<br>**Reverse Sequence**
STAT3 human	Exon 3	ACATGCCACTTTGGTGTTTCATAA<br>CAGTATAGCCGCTTCCTGCAAGAGTCGAA<br>TCTTCGTAGATTGTGCTGATAGAGAAC
STAT3 human	Exon 8	GAAGAGGCGGCAACAGATTG<br>CTGCATTGGAGGCCCGCCC<br>TTCTAGCCGATCTAGGCAGATGT
STAT3 human	Exon 22/23	AAGTTTATCTGTGTGACACCAACGA<br>TGCCGATGTCCCCCCGCA<br>CTTCACCATTATTTCCAAACTGCAT
STAT3 human	Intron 3	GCAGAGTCGGGTGTTAGTGTTCT<br>TCCTGGAAGCATCTCTTTTCTCATTTGGC<br>GCTCACGGGTAAGTATACAGAGCTT
STAT3 human	Intron 5	GCCAACAGGGAGCCTTCTCT<br>CAGGTCCTGCATGTAT<br>CAAATGAAGCCAAAACCTCAAAA
STAT3 human	Intron 6	AAGTGAGTTTGTGTTGTTGAGTTGAA<br>ACTCATTTGTCTTAACTCTG<br>CCGCCCGCCTTAAGATCTA
STAT3 human	Intron 21	TGCAGTGCCTTCTTTCACATG<br>CATCATGCTCTCTGATCCCTCAGGTTCTGT<br>GGGTGAGGTGGGCTGAGA
STAT3 human	Intron 23/Exon 24	GCAGAGGGTGGACAACTGAAC<br>AGTTTTCCCTGTCTGTCCCTCCAGAGTCC<br>GAGGTCAACTCCATGTCAAAGGT
STAT3 human	Exon 5/7 junction	GAGCAGCACCTTCAGGATGTC<br>AGAGTGCAGACATGC<br>CTGCTGCATCTTCTGCCTGGTCACT
Stat3 mouse	Exon 3	GCCACGTTGGTGTTTCATAATCT<br>TTGGGTGAAATTGACCAGCAATATAGCCG<br>GATAGAGGACATTGGACTCTTGCA
Stat3 mouse	Exon 5/7 junction	GGAGCAGCATCTTCAGGATGTC<br>CGAGTGCAGACATGCA<br>GCTGCTGCATCTTCTGTCTGGTCAC
Sod1 mouse	Exon 1	TTTTTTGCGCGGTCCTTTC<br>CGCCTTCCGTCCGTCGGCT<br>GAGGGACCAGAGAGAGCAAGAC
Sod1 mouse	Exon 3/4	CGGATGAAGAGAGGCATGTTG<br>AGACCTGGGCAATGTGACTGCTG<br>CATTGGCCACACCGTCCTTT
Sod1 mouse	Exon 5	TTGGGATTGCGCAGTAAACA<br>TCCCTGTGTGGTCTGAG<br>TTTGAGGGTAGCAGATGAGTCTGA
Sod1 mouse	Exon 1/3 junction	CTTCGAGCAGAAGGCTGTA<br>ACTCTAAGAAACATGGTGGCCCGG<br>TCACATTGCCCAGGTCTC
Sod1 mouse	Exon 2/4 junction	GACAATACACAAGGCATGTTG<br>AGACCTGGGCAATGTGACTGCTG<br>CATTGGCCACACCGTCCTTT
HNRNPH1 human	Exon 13	GAGCAGTGAACAGCAGCTACTACAG<br>CCGTGCATCTATGGGCGTGAACG

		TGACCAAGAGTCAGTGATCAGGAT
HNRNPH1<br>human	Exon 3/5<br>junction	GGATGTAGCAAGGAAGAAATTGTTC<br>TCTTCTCAGGTATATTGAAATC<br>CGTGGTGGATCATAATGAGTTCTAA
UPF1<br>human	Exon 18/19	GCCCTGACCAGAGCAAGGTA<br>CGTCATCATTGTGGGCAACCC<br>CTCCTTATAGTAGTTCAGCAGGTGGTT
Upf1<br>mouse	Exon 2/3	CGAGGAAGATGAAGAGGACACAT<br>CTACACTAAGGACCTCCCAGTCCACGCC<br>GGATCATGGATTCCACAGTAACTG
XRN1<br>human	Exon 1/2	GCCCTGACTGGGATTAGTGTTG<br>CAGAGCGGTATCCCTGTCTCAGCGA<br>CATATCCAGGTACAAGTTGTCAAATTC
Xrn1<br>mouse	Exon 3/4	TTCCTACAGAAGCCAGATTCG<br>CCAACTGTATTACGCCAGGGACTGAA<br>CACCTTGCCATGATTTGTCTG
XRN2<br>human	Exon 1	ATGGGAGTCCCGGCGTT<br>CCGCTGGCTCAGCCGCAAG<br>CCACGCAGTTGACTATGATGGA
Xrn2<br>mouse	Exon 1/2	AAGTACCCGTCCATCATTGTC<br>CCGTTGCATTCTTTTGGCTTCTCCT<br>GGATTAGGTTTACTGGCATCAAC
S*MG*6<br>human	Exon 17/18	AGTCTTCAATCACCACATCCTC<br>AGTCGTGTTCCCTCTTGGCTTCC<br>GTGGAAAGTATGTGTCAGTGG
Smg6<br>mouse	Exon 1/2	GAGCGTGTGCGGATCTC<br>TCCTTCATGTTTTCTCTGCTCCCGG<br>CCTATTATCTTTGCGCTGTCT
MALAT1<br>human		GAATTGCGTCATTTAAAGCCTAGTT<br>ACGCATTTACTAAACGCAGACGAAAATGGA<br>TCATCCTACCACTCCCAATTAATCT
Malat1<br>mouse		TGGGTTAGAGAAGGCGTGTACTG<br>CGTTGGCACGACACCTTCAGGGACT<br>TCAGCGGCAACTGGGAAA
RN7SL1<br>human/mouse		TTCACCCCTCCTTAGGCA<br>CCGGGAGGTCACCATATTGATGCC<br>CTGGAGGATCGCTTGAGTC
Gapdh<br>mouse	Exon 4	GGCAAATTCAACGGCACAGT<br>AAGGCCGAGAATGGGAAGCTTGTCATC<br>GGGTCTCGCTCCTGGAAGAT

Supplemental Table 5: Antibody information

Primary Antibody			Secondary Antibody		
**Antibody**	**Incubation**	**Vendor**	**Antibody**	**Dilution Incubation**	**Vendor**
STAT3 human	1:1000 4°C, O/N	Cell Sig. #79D7	Goat anti-rabbit IgG-HRP	1:2500 1 hour, RT	Santa Cruz sc-2030
HNRNPH1 human	1:1000 4°C, O/N	Abcam ab154894	Goat anti-rabbit IgG-HRP	1:2500 1 hour, RT	Santa Cruz sc-2030
Sod1 mouse	1:2000 4°C, O/N	Abcam ab16831	Goat anti-rabbit IgG-HRP	1:2500 1 hour, RT	Santa Cruz sc-2030
XRN1 human	1:500 4°C, O/N	Santa Cruz sc-165985	Goat anti-mouse IgG-HRP	1:2500 1 hour, RT	Abcam ab6789
Xrn1 mouse	1:5000 4°C, O/N	Bethyl A300-443A	Goat anti-rabbit IgG-HRP	1:2500 1 hour, RT	Santa Cruz sc-2030
XRN2 human	1:500 4°C, O/N	Santa Cruz sc-365258	Goat anti-mouse IgG-HRP	1:2500 1 hour, RT	Abcam ab6789
Xrn2 mouse	1:2000 4°C, O/N	Bethyl A301-103A	Goat anti-rabbit IgG-HRP	1:2500 1 hour, RT	Santa Cruz sc-2030
SMG6 human mouse	1:500 4°C, O/N	Abcam ab87539	Goat anti-rabbit IgG-HRP	1:2500 1 hour, RT	Santa Cruz sc-2030
UPF1 human mouse	1:500 4°C, O/N	Cell Sig. #9435	Goat anti-rabbit IgG-HRP	1:2500 1 hour, RT	Santa Cruz sc-2030
U1A human	1:1000 4°C, O/N	Abcam ab55751	Goat anti-mouse IgG-HRP	1:2500 1 hour, RT	Abcam ab6789
Histone H3 human	1:10000 4°C, O/N	Abcam ab1791	Goat anti-rabbit IgG-HRP	1:2500 1 hour, RT	Santa Cruz sc-2030
Calnexin human	1:1000 4°C, O/N	Abcam ab22595	Goat anti-rabbit IgG-HRP	1:2500 1 hour, RT	Santa Cruz sc-2030
α-tubulin human mouse	1:8000 4°C, O/N	Sigma T5168	Goat anti-mouse IgG-HRP	1:2500 1 hour, RT	Abcam ab6789
